# COMPARISON OF PROPOLIS EFFECTS ON TUMOR NECROSIS FACTOR ALPHA AND MALONDIALDEHYDE BETWEEN INHALATION AND CUTANEOUS ANTHRAX ANIMAL MODELS

**DOI:** 10.21010/Ajid.v16i1.1

**Published:** 2021-12-21

**Authors:** Dhani Redhono Harioputro, Wisnu Sanjaya, Yulyani Werdiningsih

**Affiliations:** 1 Department of Internal Medicine, Faculty of Medicine, Universitas Sebelas Maret, Surakarta, Indonesia

**Keywords:** anthrax, ethanolic extract of propolis, TNF-α, MDA

## Abstract

**Background:**

Inflammatory response and oxidative stress can be found in anthrax characterized by increased level of serum Tumor Necrosis Factor Alpha (TNF-α) and Malondialdehyde (MDA). The use of antibiotics in anthrax has been known to cause some disturbing side-effects, such as allergic reaction, nausea, vomiting, and antibiotic resistance. Thus, ethanolic extract of propolis (EEP) might be the alternative regimen, due to its anti-inflammatory and antioxidant properties. This study aimed to compare the effects of ethanolic extract of propolis (EEP) on TNF-α and MDA between the inhalation and cutaneous anthrax animal model.

**Materials and Methods:**

This was an experimental study with a post-test-only control group design on 40 samples of *Rattus norvegicus*. Samples were randomized into 5 groups: control, inhalation anthrax model, inhalation anthrax model + EEP, cutaneous anthrax model, and cutaneous anthrax model + EEP. After 14 days, the level of TNF-α and MDA were measured. To compare the data, we used the *ANOVA* test continued by the *post-hoc Turkey test*.

**Results:**

The results obtained showed that the level of TNF-α and MDA between the inhalation and cutaneous anthrax animal models treated with EEP were statistically different (p < 0.05). The P5 group showed the lowest level of TNF-α (6.822 ± 0.383 pg/ml) and MDA (2.717 ± 0.383 nmol/ml).

**Conclusion:**

EEP has a better effect on reducing TNF-α and MDA in cutaneous anthrax animal models compared to the inhalation anthrax animal model.

## Introduction

Anthrax is a zoonotic infectious disease caused by Bacillus anthracis. This disease is transmitted by spores via the skin, respiratory tract, or gastrointestinal tract (Martin J, et al., 2019 and Savransky V, et al., 2020). After the spore’s entrance, the vegetative form of B. anthracis develops and thereafter produces toxins. The initial responses of the infection are the production of reactive oxygen species (ROS) that can be assessed by serum malondialdehyde (MDA) level and the expression of the pro-inflammatory cytokines, such as tumor necrosis factor-alpha (TNF-α), interleukin 1β (IL-1β), IL-18, and IL-6 (Ayala et al., 2014 and Cherian DA, et al., 2019).

Cutaneous anthrax is the most common manifestation of anthrax and can be recognized early so the treatment is almost always effective. Other forms of anthrax, such as gastrointestinal and inhalational anthrax, present with very non-specific early symptoms, causing more advanced disease with relatively high mortality and morbidity (Hicks et al., 2012). The mortality rate of cutaneous anthrax without proper antibiotic treatment is 20%, while gastrointestinal anthrax is 25 - 75% and inhalation anthrax could reach 80% or higher (Kamal et al., 2011). The use of antibiotics as the treatment of anthrax has some side effects, for example, allergic reaction, nausea, vomiting, and antibiotic resistance. Thus, an alternative regimen from herbal composition might be considered.

Propolis refers to resin substances collected by the bees from various plants (Pasupuleti et al., 2017). This substance contains flavonoid that inhibits the production of some inflammatory markers, including Nitric Oxide (NO), Interleukin-1 (IL-1), IL-6, C-Reactive Proteins (CRP), and TNF-α (Zhao et al., 2016). Propolis also reduces the level of MDA by the action of Caffeic Acid Phenethyl Ester (CAPE) (Prasetyo et al., 2019). Because of these anti-inflammatory and antioxidant properties, propolis needs to be studied to compare its effect on reducing the markers of inflammation and oxidative stress on anthrax between inhalation and subcutaneous injection models.

## Materials and Methods

### Study Design

This study was an experimental research on 40 samples of *Rattus norvegicus*, using a post-test-only control group design.

### Population and Sample

The population of this study was male white rats (*Rattus norvegicus*) obtained from the Center of Inter-University Biotechnology Laboratory, Gajah Mada University, Yogyakarta, Indonesia. Samples were chosen by purposive sampling, and the intervention was decided by randomization. The sample size was counted, and 8 samples per group were used in this study. The inclusion criteria were rats aged 3 - 4 months, weighted 175–200 grams, and healthy (have shining eyes, no dull hairs, active, and good appetite). The exclusion criteria were samples that showed some signs of sickness.

### Data and Sources of Data

A total of 40 samples were randomized and placed into 5 groups: control, inhalation anthrax model, inhalation anthrax model + EEP 200 mg, cutaneous anthrax model, and cutaneous anthrax model + EEP 200 mg. The level of TNF-α and MDA were measured on the 14^th^ day.

### Statistical Analysis

Data were analyzed using SPSS 25.0 for Windows with a p-value <0.05 was considered statistically significant. To compare the effects of EEP on inhalation and cutaneous anthrax model, we used the *ANOVA* test continued by the *post-hoc Turkey* tests.

### Ethical Clearance

This study was approved by Health Research Ethics Committee, Faculty of Medicine, Universitas Sebelas Maret, no. 015/UN27.06.6.1/KEPK/EC/2020 on February 5^th^, 2020.

## Results and Discussion

### Samples Characteristic

The study used 40 rats that were divided into different groups (as shown in Table 1): 8 rats were normal, 16 rats had inhaled spores at dose 4x10¹ CFU (8 rats received no treatment, 8 rats received EEP 200 mg) and 16 rats had been injected with 0.2 cc spores subcutaneously or approximately 2x10^11^ CFU (8 rats received no treatment, 8 rats received EEP 200 mg).

**Table 1:** Samples Characteristic

Group	n	Condition	Treatment
P1	8	Normal	No treatment
P2	8	Inhaled spores at dose 4x10^10^ CFU	No treatment
P3	8	Inhaled spores at dose 4x10^10^ CFU	EEP 200 mg
P4	8	Injected spores at dose 2x10^11^ CFU	No treatment
P5	8	Injected spores at dose 2x10^11^ CFU	EEP 200 mg

### Effects of EEP in TNF-α

The level of the TNF-α on the groups were measured on the 14^th^ day as shown in Table 2. The level of TNF-α from the highest to the lowest level were consecutively P3 group (14.282 ± 0.282 pg/ml), P4 (14.247 ± 0.311 pg/ml), P2 (14.192 ± 0.404 pg/ml), P5 (6.822 ± 0.383 pg/ml) and the lowest level found in the P1 (6.056 ± 0.394 pg/ml). All of the groups with anthrax showed a higher level of TNF-α compared to the control group. Between those anthrax groups, the group with cutaneous model receiving EEP 200 mg had the lowest level of TNF-α.

**Table 2:** Results of TNF-α measurement

Group	n	Mean	SD	p
P1	8	6.056	0.394	0.001
P2	8	14.192	0.404	
P3	8	14.282	0.282	
P4	8	14.247	0.311	
P5	8	6.822	0.383	

**Figure 1:**
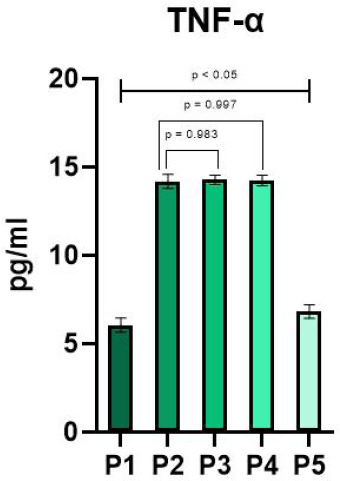
Results of the ANOVA and post-hoc Turkey tests of Serum TNF-α Level

The ANOVA test continued by *post-hoc Turkey* test was performed. The results were summarized in Figure 1. Every 2 groups compared showed significant difference (p < 0.05), except the P2-P3 (p = 0.983) and P2-P4 (p = 0.997). The effect of EEP in the inhalation model and cutaneous model was significantly different (P3-P5, p<0.05), with the effect of EEP in the cutaneous anthrax animal model being better.

### Effects of EEP on MDA

The level of MDA was measured on the 14^th^ day as seen in Table 3. From the highest to the lowest level of MDA between groups were consecutively P1 (11.287 ± 1.904 nmol/ml), P3 (9.866 ± 0.220 nmol/ml), P4 (9.642 ± 0.383 nmol/ml), P2 (9.533 ± 0.246 nmol/ml), and the lowest level found in P5 (2.717 ± 0.383 nmol/ml). Surprisingly, the control group showed the highest level of MDA. Between all of the anthrax groups, P5 showed the lowest level of MDA.

**Table 3:** Results of MDA measurement

Group	N	Mean	SD	p
P1	8	11.287	1.904	0.001
P2	8	9.533	0.246	
P3	8	9.866	0.220	
P4	8	9.642	0.383	
P5	8	2.717	0.383	

**Figure 2:**
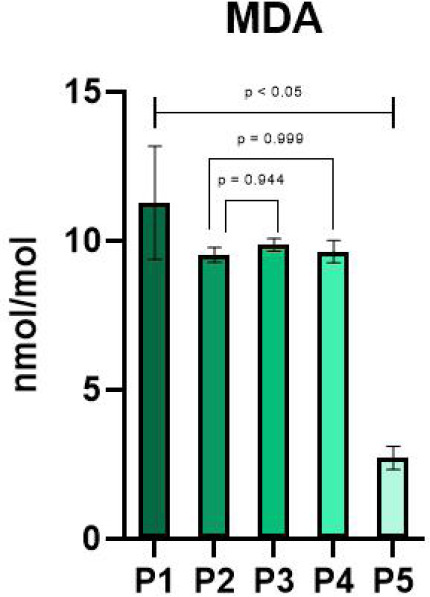
Results of the ANOVA and post-hoc Turkey tests of Serum MDA Level

The statistical analysis was performed using the ANOVA test continued by *post-hoc Turkey* test. The results can be seen in Figure 2 below. Every 2 groups compared showed significant difference (p < 0.05), except the P2-P3 (p = 0.944) and P2-P4 (p = 0.999). The effect of EEP in the inhalation model and cutaneous model was significantly different (P3-P5, p<0.05), with the effect of EEP on the level of MDA in cutaneous anthrax animal model was better.

## Discussion

This is an experimental study aimed to compare the effect of EEP 200 mg on the level of TNF-α and MDA between the rat model of inhalation and cutaneous anthrax after 14 days. Inhaled or injected spores induce an inflammatory response, marked by TNF-α. This response might develop into oxidative stress where Reactive Oxygen Species (ROS) are produced. This oxidative stress could be assessed by measuring the serum MDA level (Ayala et al. 2014 and Cherian et al., 2019). EEP has been known to reduce the inflammatory response and oxidative stress (Hori et al., 2013 and Pahlavani et al., 2020). Thus, EEP might have some positive effects on anthrax. Kamal (2011) stated because the inhalation anthrax has higher morbidity and mortality compared to cutaneous anthrax, the effect of EEP on these 2 forms of anthrax needs to be assessed.

The findings of this study showed that all of the anthrax groups had a higher level of TNF-α and lower level of MDA compared to the control group. P5 had the lowest level of TNF-α between the anthrax groups (6.822 ± 0.383 pg/ml) and also the lowest level of MDA (2.717 ± 0.383 nmol/ml). Comparison of EEP given to the inhalation and cutaneous anthrax model also showed significant difference (p < 0.05), with the better effect shown in the cutaneous anthrax model.

The study revealed that injection of EEP also resulted in considerably decreased serum E-selectin, SGPT, and creatinine levels (Redhono et al., in press). The positive effect of EEP in this study is relevant to a previous study in rat models of cutaneous anthrax which showed no cutaneous manifestation after EEP was given (Kusumawardani et al., 2020). The absence of lesion proved that there was only minimal inflammatory reaction happened, thus oxidative stress would be minimized too. In another study with rat models of inhalation anthrax, EEP was shown to reduce the inflammatory response in the lung tissue and also showed its antioxidant effect on reducing ROS production (Redhono et al., in press).

The comparison that showed a better effect on the cutaneous model than on the inhalation model might be due to the severity of those 2 forms of anthrax. Cutaneous anthrax appears primary as a papule that progresses to a large vesicle that may develop an eschar if ruptured. Study conducted by Redhono et al. (2018), showed that clinical symptoms in the form of eschar are seen in 11.9 percent of patients with cutaneous anthrax. Patients with cutaneous anthrax may have a resolution of illness or develop signs of disseminated disease, but the risk of dissemination and death can be minimized by treating the patient with antibiotics. While inhalation anthrax is the most lethal form of anthrax with non-specific signs and symptoms, for example, malaise, fever, and nonproductive cough. It may cause the delay of diagnosis and treatment, and the worst of it is death usually results within 72 hours after symptoms start (Chambers et al., 2021).

## Conclusion

Based on the results of this study, EEP has a better effect on reducing the serum level of TNF-α and MDA as markers of inflammation and oxidative stress in the cutaneous anthrax animal models.

### Conflict of Interest

The authors declare that there is no actual or potential conflict of interest concerning this study.

**List of Abbreviations:**
Cape: caffeic Acid Phenethyl Ester
CRP: C-Reactive Protein
EEP: Ethanolic Extract of propolis
IL: Interleukin
MDA: Malondialdehyde
NO: Nitric Oxide
ROS: Reactive Oxygen Species
TNF: Tumor Necrosis Factor
